# Synchronization bandwidth enhancement induced by a parametrically excited oscillator

**DOI:** 10.1038/s41378-024-00709-1

**Published:** 2024-07-08

**Authors:** Jiahao Song, Yutao Xu, Qiqi Yang, Ronghua Huan, Xueyong Wei

**Affiliations:** 1 State Key Laboratory for Manufacturing Systems Engineering, Xi’an Jiaotong University, Xi’an, 710049 People’s Republic of China; 2 Department of Mechanics, Zhejiang University, Hangzhou, 310027 People’s Republic of China; 3 School of Instrument Science and Technology, Xi’an Jiaotong University, Xi’an, 710049 People’s Republic of China

**Keywords:** Electrical and electronic engineering, Sensors

## Abstract

The synchronization phenomenon in nature has been utilized in sensing and timekeeping fields due to its numerous advantages, including amplitude and frequency stabilization, noise reduction, and sensitivity improvement. However, the limited synchronization bandwidth hinders its broader application, and few techniques have been explored to enhance this aspect. In this paper, we conducted theoretical and experimental studies on the unidirectional synchronization characteristics of a resonator with phase lock loop oscillation. A novel enhancement method for the synchronization bandwidth using a parametrically excited MEMS oscillator is proposed, which achieves a remarkably large synchronization bandwidth of 8.85 kHz, covering more than 94% of the hysteresis interval. Importantly, the proposed method exhibits significant potential for high-order synchronization and frequency stabilization compared to the conventional directly excited oscillator. These findings present an effective approach for expanding the synchronization bandwidth, which has promising applications in nonlinear sensing, fully mechanical frequency dividers, and high-precision time references.

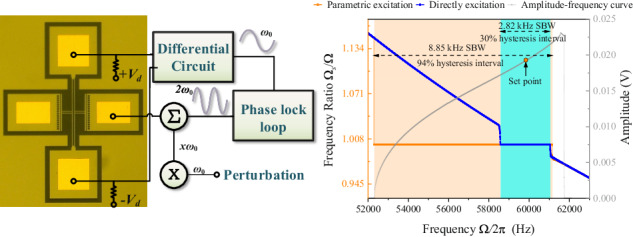

## Introduction

Synchronization, a universal and natural phenomenon, was first observed in the Huygens clock in the 17th century and subsequently discovered in other fields, such as living organisms^1,2^, the internet^3^, and celestial bodies^4^. Synchronization occurs between self-sustaining oscillation systems with weak coupling, which can be unidirectional^5–7^ or interactive^8–11^. For unidirectional synchronization, an external weak signal is injected into a self-sustained oscillation system; when the signal lies in a certain frequency range, the oscillation frequency is entrained by the external signal^12^. Therefore, unidirectional synchronization is also known as injection locking. In MEMS fields, synchronization shows prominent improvements in frequency stability^8,13,14^, phase noise reduction^5,9,15–17^, and amplitude stabilization^7^. Moreover, by designing resonators that meet superharmonic synchronization conditions, both the frequency readout and stability can be multiplied^18–20^. Therefore, synchronization is an effective technical approach for improving the performance of MEMS sensors and frequency reference devices.

However, the synchronization bandwidth determines the range of device performance enhancements and has become the principal issue in synchronous-based applications. Because N/MEMS devices are prone to nonlinearity effects and superior control^10,21–24^, self-sustaining oscillators with Duffing nonlinearity have been demonstrated to significantly enhance the synchronized region^6^. In many nonlinear stiffness adjustment methods, such as shape optimization^25–28^, Joule heating^29,30^, dispersive coupling^23,31^, and preloaded strain^32^, the electrostatic force is an effective approach for generating negative cubic nonlinearity^33,34^. Therefore, it has been used to improve the SBW by 1~2 times through the superposition of the softening nonlinearity of the curved beam itself and the negative electrostatic stiffness^35^. Based on nonlinear MEMS resonators, Xu et al. developed an automatic frequency tracking device based on PID regulation and integrated it into a MEMS resonant accelerometer to tune a synchronous system suitable for wide-range measurements^36^. Furthermore, by setting the optimal PID parameters, the relocking time can be reduced by approximately 100 ms as the SBW increases to 1.2 kHz^37^. Nevertheless, this technique of tracking the frequency of the external circuit undoubtedly increases the manufacturing cost of the sensor. Moreover, the response time of a PID lies at the core of the application of this method in the fields of rapid detection and real-time feedback control, which brings additional design difficulties. In addition, by exploring the inherent characteristics of self-sustaining oscillators, it has been demonstrated that the injection-locking SBW can be enhanced to a large relative bandwidth exceeding 60% in a silicon optomechanical crystal cavity^38^.

Parametric resonance is a type of self-excited vibration generated by periodic variations in the system itself when the excitation frequency is 2/*n* times the natural frequency of the vibration system. In micro/nanomechanical systems, parametric resonance has been demonstrated to have potential advantages, such as noise suppression^39–41^ and signal amplification^42,43^. Although synchronization characteristics have been extensively studied in directly excited self-sustaining systems, to our knowledge, synchronization with parametrically excited oscillation has rarely been discussed, and few approaches for enhancing synchronous bandwidth have been reported.

In this paper, we introduce a method for generating a giant synchronization bandwidth via a parametrically excited MEMS resonator. Unidirectional synchronization characteristics in parametric excitation systems have been studied in detail for the first time, demonstrating a significant improvement in SBW compared to directly excited self-sustained oscillation. A theoretical model for predicting the SBW is established that includes nonlinear damping and cubic and quintic nonlinear stiffness. With a weak perturbation signal, the synchronization bandwidth of a parametrically excited resonator can reach 8.85 kHz, which covers a 94% hysteresis interval and exhibits great potential for improving subharmonic synchronization and frequency stability. The proposed method contributes to overcoming the bottleneck that synchronization is difficult to apply in engineering fields due to bandwidth constraints.

## Results and discussion

### Dynamic response subjected to parametric excitation

The length, width, and thickness of a double-end tuning fork fabricated by the standard silicon-on-insulator process are 416 μm × 3.4 μm × 25 μm. The protruding plates on the resonant beam are designed to form electrostatic forces with the fixed electrodes. Both ends of the resonant beam and the fixed electrodes are anchored on a silicon substrate, and gold is sputtered on the surface to connect the external test circuit. The detailed geometric parameters and basic characteristics are given in Table S1.

**Fig. 1:**
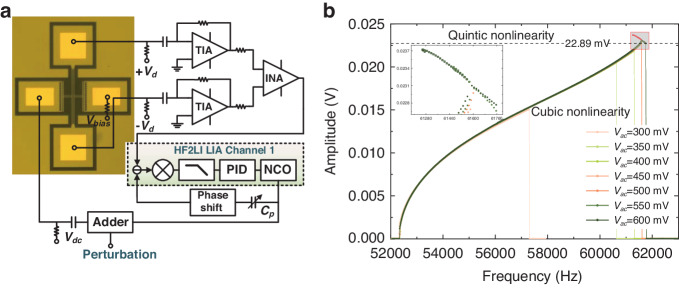
**The experimental setup and dynamic response. a** *V*_*d*_ = ±10 V was applied to the ends of the resonator to ensure that the protruding driving electrodes were at 0 V. Because the silicon material itself has piezoresistive properties, its resistance changes as the resonator vibrates. The dynamic current is amplified by a TIA and then differentially divided to obtain the amplitude of the resonator. The perturbation signal was generated from HF2LI channel 2, and *V*_*bias*_ was set to 20 V. **b** Parametrically excited dynamic response with *V*_*ac*_ from 300 mV to 600 mV and *V*_*dc*_ = 35 V. The squares in the subgraph are measured by tuning the phase delay in the PLL module built in HF2LI

The resonator is placed in a vacuum chamber (<0.1 Pa) with a phase lock loop (PLL) built in a locked-in amplifier HF2LI, as shown in Fig. 1a. Using *V*_*ac*_ = 550 mV and *V*_*dc*_ = 35 V to ensure that the driving force exceeds the critical value to build parametric oscillation^44,45^, the natural frequency *ω*_0_ of the resonator is 52.86 kHz with a hysteresis interval of 9.32 kHz. Because the excitation frequency is twice the demodulation frequency, the parasitic feedthrough does not generate responses at the demodulation frequency; thus, a pure resonator motion signal can be obtained^46^. The backbone curve can be described as $\varOmega ={\omega }_{0}+\frac{3{A}^{2}\beta }{8{\omega }_{0}}+\frac{5{A}^{4}\kappa }{16{\omega }_{0}}$ (see Eq. (S8) in the supplementary materials), where the frequency is dominated by two polynomials with respect to the amplitude. When the amplitude of the resonator reaches $\sqrt{-\frac{6\beta }{5\kappa }}$, the quadratic and quartic terms on the frequency contribute equally. The effects of nonlinear terms on the frequency cancel each other, and the frequency is decoupled from the amplitude. As Fig. 1b shows, the resonator exhibits a hardening third stiffness characteristic (*β* > 0) before the decoupling point. As the amplitude increases, the effect of quintic nonlinearity gradually dominates, and the resonator exhibits stiffness-softening characteristics (*κ* < 0).

### Unidirectional synchronization characteristics

**Fig. 2:**
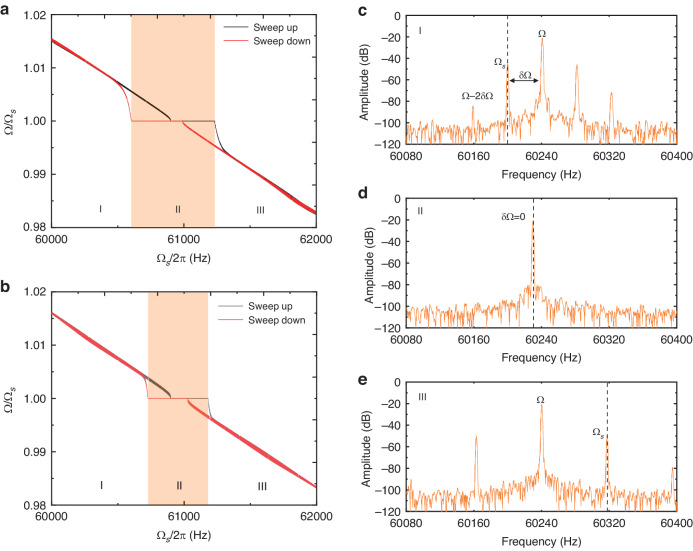
**Unidirectional synchronous characteristics**. With *V*_*ac*_ = 550 mV for parametric excitation and *V*_*ac*_ = 335 mV for direct excitation, the self-sustained oscillation is locked at an amplitude of 20.4 mV. The perturbation strength is maintained at 10 mV. **a** Parametric excitation. **b** Direct excitation. **c**–**e** Spectral response of the resonator in three states during synchronization (I before synchronization, II synchronized, III synchronization loss). The perturbation frequency is swept from 60 kHz to 61 kHz

Unidirectional synchronization experiments were performed with direct perturbation (*f*_*s*_cos(Ω_*s*_*t* + *φ*)). To eliminate the static feedthrough signal of resonators during direct excitation, an adjustable capacitor (1~10 pF) was employed, as illustrated in Fig. 1a. A phase shifter was used to adjust the phase of the adjustable capacitor to be consistent with the parasitic feedthrough of the resonator, and the size of the adjustable capacitor was tuned to match the amplitude of the feedthrough signal. By using the differential input mode, the pure motion signal of the resonator can be obtained. The self-sustained oscillation system was built with a phase-lock loop (PLL). The phase of the resonator can be obtained by multiplying the input resonator motion signal and the numerically controlled oscillator (NCO) signal. The multiplied high-frequency signal can be filtered out by a low-pass filter (LPF) to retain the phase information of the resonator. The PID was used to control the output of the NCO based on the phase change of demodulation to form a closed-loop oscillation of the resonator. With the target bandwidth set to 30 Hz, the resonator oscillation amplitude can be precisely adjusted to 20.4 mV by tuning the phase delay. The external tune was set away from the frequency of the self-sustained system, and then the external tune was swept upward and downward. The unidirectional SBW can be obtained, as shown in Fig. 2a. Figure 2b shows the synchronization characteristics of the directly excited oscillator. The two excitation modes exhibit the same characteristic in the spectrum, corresponding to three different states, as shown in Fig. 2c–e. When the perturbation frequency is far from the oscillation frequency Ω, the external tune and the resonator oscillate independently with the appearance of harmonic sidelobes. Then, the perturbation frequency approaches the main spectrum peak of the resonator, the sidelobe and the main frequency peak are reconciled, and synchronization occurs. As the disturbance frequency continues to increase, when Ω_*c*_ cannot compensate for ΔΩ = 0, which follows Eq. (10), the self-sustained oscillation and the external tuning lose synchronization and return to an independent oscillation state.

### Synchronization bandwidth enhancement

To demonstrate the significant intensification of the parametric oscillation on the SBW, we compared the relationship between the SBW and oscillation amplitude with different driving modes. We adjusted the maximum amplitude of the two driving modes to be consistent. Then, the SBW of the resonator with a parametrically excited oscillator and directly excited oscillator at different amplitude points was probed by locking the phase delay while considering a weak perturbation signal *V*_*ac*_ = 10 mV. During the measurement of the SBW, we first adjusted the frequency of the external signal to have a sufficiently small frequency mismatch with the oscillation frequency to construct a synchronization state. Then, the frequency of the external signal was swept forward and backward to obtain a precise SBW. Compared to direct frequency scanning of external signals over a wide range, in which the self-sustained oscillation and external tuning are vibrated independently, the measurement method that builds the synchronous state first is more in line with the actual situation^36,47^.

**Fig. 3:**
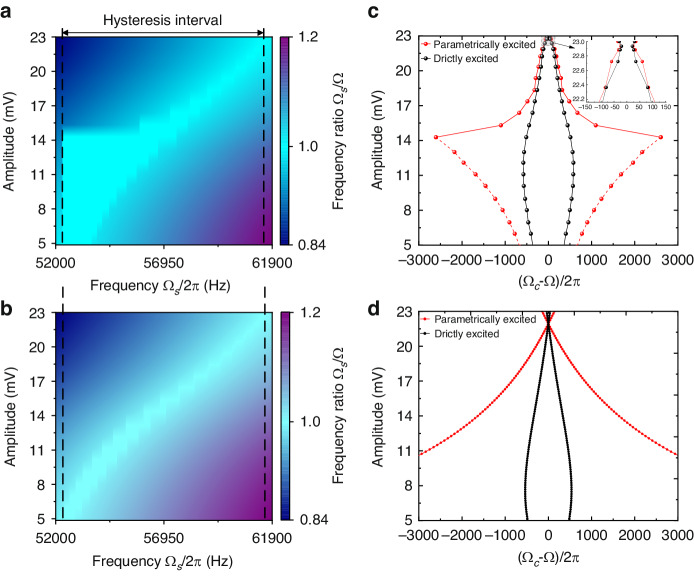
**Synchronization bandwidths of different amplitudes**. To ensure consistent cubic and quintic nonlinear stiffness, the resonator was excited to have the same maximum amplitude under different driving modes by adjusting the AC excitation voltage. The DC voltage *V*_*dc*_ was maintained at 35 V. The AC excitation voltage was found to be 550 mV for parametric excitation and 335 mV for direct excitation. The frequency ratio Ω_*s*_/Ω versus the amplitude of the self-sustaining oscillation is depicted using a colormap plot, where the cyan-colored region is synchronized. **a** Parametric excitation. **b** Direct excitation. The SBW with respect to the amplitude with **c** experimental results and **d** theoretical results following Eqs. (12), (13)

Figure 3 presents the experimental and theoretical results demonstrating the SBW of PLL oscillators with two different excitation scenarios with varying amplitudes and validating the improvement of the parametric excitation. The nonlinear parameters of the resonator are determined by tuning the driving strength. As predicted by the theoretical model, the SBW shows a trend of initially increasing and then decreasing with increasing amplitude in the directly excited oscillator. Until the 3rd and 5th nonlinear interactions cancel each other, the bandwidths almost decrease to zero, leaving only the contribution of the linear term. Subsequently, the SBW increases in the quintic nonlinear dominant regime. In contrast to the directly excited oscillator, the smaller the amplitude of the parametrically excited oscillation at a certain excitation force *f*_0_ is, the larger the SBW. However, when the SBW exceeds the hysteresis interval of the resonator, the resonator cannot maintain self-sustained oscillation. Therefore, the SBW of the parametrically excited oscillation decreases after the synchronous state reaches the hysteresis interval boundary of the resonator. The error of the theoretical results mainly stems from the parameter identification of the vibration system, and the theoretical model does not consider the influence of the hysteresis interval; thus, the bandwidth monotonically increases with decreasing amplitude. By introducing parametric oscillation, the SBW is enhanced by 448% compared to that of the classical directly excited oscillator under a weak perturbation strength of 10 mV. It is worth noting that the SBW is highly related to the hysteresis interval of resonators. In practical applications, the resonator structure can be reasonably designed to obtain the required bandwidth^25,48^. Moreover, comparing the SBW from Eqs. (12) and (13), reducing nonlinear damping is an effective way to further increase the synchronization bandwidth. Reasonable clamp^49^, electrical adjustment^50^, and temperature control^51^ designs have been demonstrated to be able to adjust nonlinear damping and may be used to further extend SBWs.

**Fig. 4:**
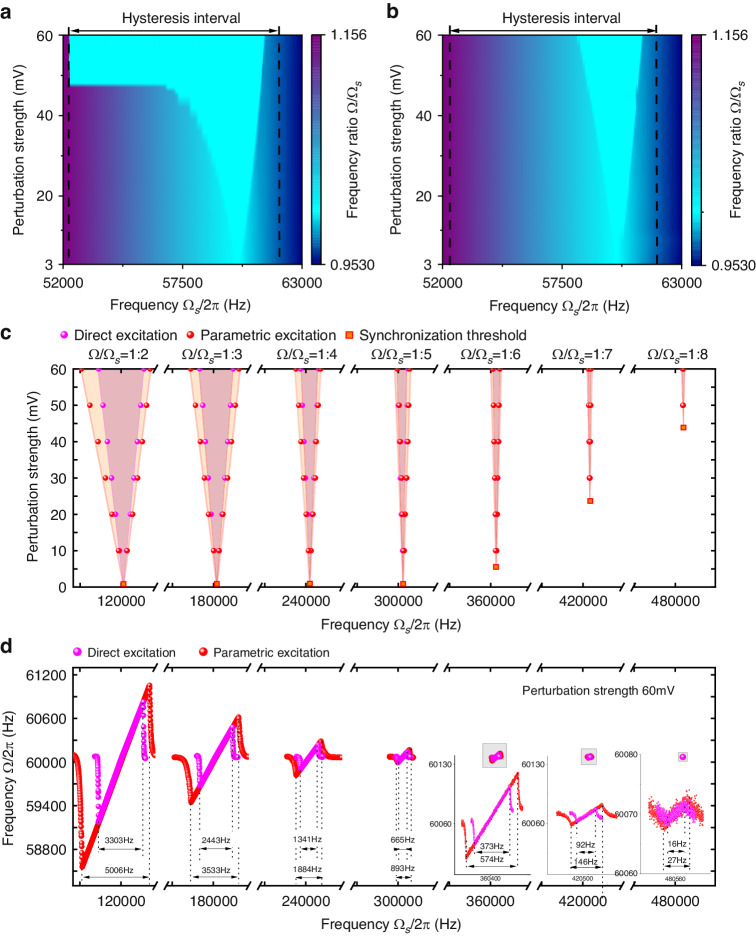
**Bandwidth amplification during high-order unidirectional synchronization**. The DC voltage *V*_*dc*_ was maintained at 35 V, and the driving voltage *V*_*ac*_ was 550 mV for the parametrically excited oscillator and 335 mV for the directly excited oscillator to actuate the same maximum amplitude. The SBW with respect to the perturbation strength of parametric excitation (**a**) and direct excitation (**b**) for perturbation strengths from 3 mV to 60 mV. **c** Arnold tongues measured from Ω/Ω_*s*_ = 1:2 to 1:8. The colored regions are the predicted SWB. **d** The variation in the frequency Ω with increasing disturbance frequency

Synchronization has been applied to sensing systems and proven to significantly improve sensor performance, where the perturbation signal represents the sensing signal; thus, the effect of perturbation strength is vital for the SBW. We considered the case in which the resonator with a parametrically excited oscillator is subjected to a direct injection signal (*f*_*s*_cos(Ω_*s*_*t* + *φ*)). To prevent excessive disturbance intensity from affecting the resonator to maintain its self-sustained state, an external tune was added with *V*_*ac*_ = 3~60 mV at the 3^rd^ dominant oscillation state. We measured the SBW with a closed-loop oscillation amplitude of 19.7 mV. As shown in Fig. 4a, the SBW increases monotonically with increasing perturbation strength until almost the entire hysteresis interval is covered, which results in an asymmetric Arnold tongue shape. Due to the hysteresis effect, the SBWs measured during upward and downward sweeping are inconsistent. The SBW of the parametrically excited oscillator is 8.85 kHz, while that of the directly excited oscillator is only 2.82 kHz, and the perturbation strength is 60 mV. Furthermore, the enhancement of a parametrically excited oscillator on an SBW under high-order unidirectional synchronization was investigated. Here, we measured the SBW from Ω/Ω_*s*_ equal to 1:2 to 1:8 under disturbance perturbation strengths from 10 mV to 60 mV, as shown in Fig. 4c. With increasing synchronization order, the SBW decreases, and the perturbation threshold increases. When synchronization reaches the order of 1:8, the synchronization phenomenon can only be observed when the disturbance strength is greater than 45 mV. Higher-order synchronization can be achieved by a higher disturbance intensity signal. In Fig. 4d, the frequency of the self-sustained oscillation is entrained and increases with the disturbance frequency. The detailed synchronization bandwidth of each order is marked. The SBW of the parametrically excited oscillator invariably improves by approximately 1.45 times compared to that of the direct excitation.

### Frequency stability improvement

**Fig. 5:**
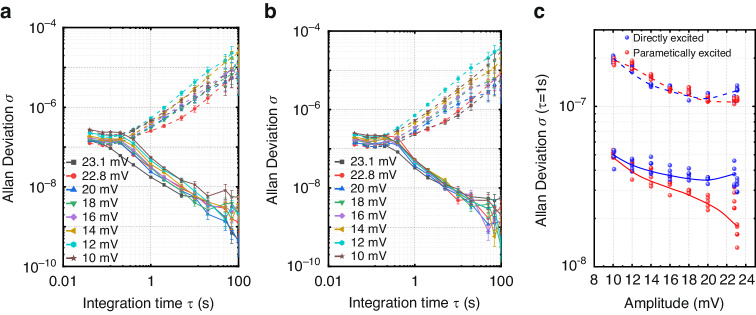
**Comparison of frequency stability**. Measured Allan deviation with different amplitudes with fixed DC voltage *V*_*dc*_ = 35 V; *V*_*ac*_ = 550 mV for parametric excitation (**a)** nd *V*_*ac*_ = 335 mV for direct excitation (**b**), where the dashed lines and solid lines are the results before synchronization and after synchronization, respectively. **c** Frequency stability comparison at an integration time of τ = 1 s; five sets of Allan deviations were measured at each amplitude. The dashed lines are the fitted results before synchronization, and the solid lines are the fitted results after synchronization

One of the important advantages of applying the synchronization phenomenon is the improvement of frequency stability^52^. Here, the Allan deviation is utilized to measure the frequency stability of the resonator with a parametrically excited oscillator and a directly excited oscillator in the synchronous state. By tuning the phase delay, we built self-sustained oscillations at peak amplitude, moderate amplitude, and low amplitude. The Allan deviation is expressed as:

1 $${\sigma }_{A}(\tau )=\sqrt{\frac{1}{2(N-1)}\mathop{\sum }\nolimits_{i=1}^{N-1}{\left(\frac{\overline{{f}_{i+1}}-{\bar{f}}_{i}}{{f}_{0}}\right)}^{2}}$$

where *τ* is the integral time, *N* is the number of frequency points collected, and $\bar{{f}_{i+1}}$ and $\bar{{f}_{i}}$ are the collected frequency data. The smaller the Allen deviation is, the smaller the frequency fluctuation during the integration time, indicating greater frequency stability of the oscillator. Figure 5a, b shows the Allan deviation of the parametrically excited oscillator before and after synchronization for 100 s at different amplitude points. When the resonator is not synchronized with the external rhythm, the frequency stability increases as it approaches the peak point because the phase slope increases. The frequency signal output by the oscillator is affected by phase noise and temperature drift. During a short integration time, the phase noise is constant; thus, the Allan deviation shows a decreasing trend with integration time. As the integration time increases, the influence of temperature drift becomes dominant, resulting in an increase in the Allan deviation, which overall shows a trend of first decreasing and then increasing. After synchronization with the external tuning, the frequency of the resonator is constrained by the external perturbation signal, and the frequency stability gradually approaches that of the external tuning with increasing integration time. To compare the two different driving scenarios, we conducted repeated experiments on the frequency stability of five sets of oscillators at different amplitude points during a 1 s integration time, as shown in Fig. 5c. Without the perturbation signal, the frequency stability of the oscillators is basically the same at different amplitudes. The frequency stability of the parametrically excited oscillator is significantly better than that of direct excitation after synchronization. At the peak amplitude, the Allan deviation of the parametrically excited oscillation is approximately 18 ppb, while that of the direct excitation is only 38 ppb. The parametrically excited oscillation not only improves the SBW but also significantly enhances the frequency stability compared to direct excitation after synchronization.

## Conclusion

In this paper, we proposed an efficient method to enhance the synchronization bandwidth based on parametrically excited oscillation. The unidirectional synchronization characteristics of parametrically excited oscillators were studied both experimentally and theoretically. With a weak perturbation strength, the effect of the SBW on different closed-loop oscillation amplitudes was studied, and the SBW was approximately 4.5 times greater than that of classic direct excitation. Furthermore, the perturbation strength and subharmonic synchronization were investigated. By increasing the disturbance intensity, we measured the asymmetric Arnold tongue of the parameter excited oscillation and achieved an 8.85 kHz SBW, which covers a 94% hysteresis interval. Moreover, high-order synchronization up to 1:8 is achieved, and the enhancement of parametrically excited oscillation during high-order unidirectional synchronization is demonstrated. The frequency stability at different amplitudes was investigated, and the experimental results show that the proposed enhancement approach based on parametrically excited oscillation exhibits better stability after synchronization than direct excitation. Notably, the required synchronization bandwidth can be achieved by adjusting the amplitude point of the closed-loop oscillation, injecting the disturbance intensity, and reasonably designing the nonlinear stiffness of the resonator. In addition to the synchronization bandwidth, the synchronization process and synchronization time are also worth exploring. Future work will focus on exploring new working mechanisms or external control modes for regulating synchronization time.

## Materials and methods

### Theoretical analysis

Resonator transduction uses the piezoresistive method, and the self-sustaining system can be modeled as:

2 $$\ddot{x}+\mu \dot{x}+\eta \dot{x}{x}^{2}+{\omega }_{0}^{2}x+\beta {x}^{3}+\kappa {x}^{5}={f}_{0}x\,\cos (2\phi (t)+{\phi }_{0})+{f}_{s}\,\cos ({\varOmega }_{s}t+\varphi )$$

where *x* is the in-plane displacement of the resonator, *μ* is the linear viscous damping, and *ω*_0_ is the first flexure mode frequency. *η*, *β*, and κ represent the dimensionless nonlinear damping, cubic stiffness and quintic stiffness, respectively. *ϕ*(*t*)=Ω*t* + *θ* describes the frequency and phase of actuation. *ϕ*_0_ is the preset phase delay. By introducing the van der Pol transformation *x*(*t*)=*A*(*t*)cos(*ϕ*(*t*)) and d*x*/d*t* = -*A*(*t*)Ωcos(*ϕ*(*t*)), the unidirectional synchronization characteristics with a parametrically excited oscillator described by Eq. (2) can be solved. Then, the following equation can be obtained

3 $$\begin{array}{ll}{\displaystyle{{A}^{\prime} (t)=\frac{{A}^{5}\kappa \,\sin (\phi ){\cos }^{5}(\phi )}{\varOmega }+\frac{{A}^{3}\beta \,\sin (\phi ){\cos }^{3}(\phi )}{\varOmega }-{A}^{3}\eta {\sin }^{2}(\phi ){\cos }^{2}(\phi )-A\varOmega \,\sin (\phi )\cos (\phi )}}\\ \qquad\qquad{\displaystyle{-\,A\mu {\sin }^{2}(\phi )+\frac{A{\omega }^{2}\,\sin (\phi )\cos (\phi )}{\varOmega }-\frac{A{f}_{0}\,\sin (\phi )\cos (2\phi +{\phi }_{0})\cos (\phi )}{\varOmega }-\frac{{f}_{s}\,\sin (\phi )\cos (\theta -\varphi -\phi )}{\varOmega }}}\\ \qquad\qquad{\displaystyle{{\theta}^{\prime} (t)=\frac{{A}^{4}\kappa {\cos }^{6}(\phi )}{\varOmega }+\frac{{A}^{2}\beta {\cos }^{4}(\phi )}{\varOmega }-{A}^{2}\eta \,\sin (\phi ){\cos }^{3}(\phi )-\mu \,\sin (\phi )\cos (\phi )+\frac{{\omega }^{2}{\cos }^{2}(\phi )}{\varOmega }}}\\ \qquad\qquad{\displaystyle{-\,\varOmega {\cos }^{2}(\phi )-\frac{{f}_{s}\,\cos (\phi )\cos (\theta -\varphi -\phi )}{A\varOmega }-\frac{{f}_{0}\,\cos (2\phi +{\phi }_{0}){\cos }^{2}(\phi )}{\varOmega }}}\end{array}$$

By averaging Eq. (3) over a period, we obtain the amplitude and phase relationship of the system as follows:

4 $${A}^{\prime} (t)=-\frac{{A}^{3}\eta }{8}-\frac{A\mu }{2}+\frac{A{f}_{0}\,\sin ({\phi }_{0})}{4\varOmega }+\frac{\sin (\vartheta ){f}_{s}}{2\varOmega }$$

5 $${\theta}^{\prime} (t)=\Delta \omega +\frac{3{A}^{2}\beta }{8\varOmega }+\frac{5{A}^{4}\kappa }{16\varOmega }-\frac{{f}_{0}\,\cos ({\phi }_{0})}{4\varOmega }-\frac{\cos (\vartheta ){f}_{s}}{2A\varOmega }$$

where $\varDelta \omega =\frac{{\omega }_{0}^{2}-{\varOmega }^{2}}{2\varOmega }\approx {\omega }_{0}-\varOmega$. Assuming that the external force *f*_*s*_ = 0, the steady-state amplitude can be obtained by considering ${A}^{\prime} (t)=0$.

6 $$R=\frac{\sqrt{2}\sqrt{{f}_{0}\,\sin ({\phi }_{0})-2\mu \varOmega }}{\sqrt{\eta }\sqrt{\varOmega }}$$

In Eqs. (4) and (5), *A* is the amplitude, and *θ* is the phase of the oscillator. Δ*ω* ≈ *ω*_0_-Ω and *ϑ* = *φ*-*θ* are the frequency and phase differences between self-sustained oscillation and external perturbation, respectively. When synchronization occurs, the frequency of the oscillator is entrained by an external excitation signal, with the frequency difference equal to zero and the phase difference remaining constant. The trajectory of the oscillator deviates from the stable limit cycle. After transient response attenuation, the trajectory remains stable near the previous stable limit cycle. Therefore, the amplitude during synchronization can be described as:

7 $$A(t)=R+\varepsilon r(t)$$

Substituting the amplitude *A*(*t*) into Eq. (4) and considering only the uncertainties caused by the disturbance, the expression for the perturbed amplitude *r*(*t*) of the disturbance can be obtained as:

8 $${r}^{\prime} (t)=\frac{{f}_{0}r(t)\sin ({\phi }_{0})}{4\varOmega }+\frac{{f}_{s}\,\sin (\vartheta )}{2\varOmega \epsilon }-\frac{3}{8}\eta {R}^{2}r(t)-\frac{3}{8}\eta {R}^{2}\epsilon r{(t)}^{2}-\frac{1}{2}\mu r(t)-\frac{1}{8}\eta {\epsilon }^{2}r{(t)}^{3}$$

After the transient response attenuation and neglecting the high-order terms in Eq. (8), we obtain

9 $$r(t)=\frac{4{f}_{s}\,\sin (\vartheta )}{3\eta {R}^{2}\varOmega \epsilon +4\mu \varOmega \epsilon -2{f}_{0}\epsilon \,\sin ({\phi }_{0})}$$

Substituting *A*(*t*) into Eq. (5) and preserving the terms up to O(*ε*), we obtain the equation of phase delay

10 $$-\dot{\vartheta }(t)=\varDelta \varOmega +{\varOmega }_{c}$$

where ΔΩ = Ω-Ω_*s*_ represents the magnitude of the deviation of the disturbance frequency from the self-excited oscillation frequency, and synchronization occurs in the range [Ω-Ω_*c*_,Ω + Ω_*c*_] with the phase difference unchanged ($\dot{\vartheta }$ = 0).

11 $${\varOmega }_{c}=-\frac{\cos (\vartheta ){f}_{s}}{2R\varOmega }+\frac{3\beta \,\sin (\vartheta ){f}_{s}}{3R\eta {\varOmega }^{2}}+\frac{5R\kappa \,\sin (\vartheta ){f}_{s}}{\eta {\varOmega }^{2}}$$

Therefore, the synchronization bandwidth under parametric excitation can be expressed as:

12 $${B}_{p}=2\sqrt{{\left(\frac{{f}_{s}}{2R\varOmega }\right)}^{2}+{\left(\frac{3\beta {f}_{s}+5\kappa {R}^{2}{f}_{s}}{2\eta R{\varOmega }^{2}}\right)}^{2}}$$

In the case of direct excitation, the synchronization bandwidth is defined as

13 $${B}_{d}=2\sqrt{{\left(\frac{{f}_{s}}{2R\varOmega }\right)}^{2}+{\left(\frac{3\beta R{f}_{s}+5\kappa {R}^{3}{f}_{s}}{4\mu {\varOmega }^{2}+3\eta {R}^{2}{\varOmega }^{2}}\right)}^{2}}$$

The derivation of Eq. (13) is presented in Supplementary S4.

### Supplementary information

Supplementary
