# Supplementary material for: Synchronization bandwidth enhancement induced by a parametrically excited oscillator
Source: Microsyst Nanoeng. 2024 Jul 8;10:95. doi: 10.1038/s41378-024-00709-1 (PMC11231294; doi:10.1038/s41378-024-00709-1)
Supplement: Supplementary file 1 — Supplementary [file 41378_2024_709_MOESM1_ESM.docx]

Supplementary materials for

Synchronization bandwidth enhancement induced by a parametrically excited oscillator

Jiahao Song^1^, Yutao Xu^1^, Qiqi Yang^1^, Ronghua Huan^3^, Xueyong Wei^1, 2, *^

^1^State Key Laboratory for Manufacturing Systems Engineering, Xi’an Jiaotong University, Xi’an 710049, People’s Republic of China

^2^School of Instrument Science and Technology, Xi’an Jiaotong University, Xi’an 710049, People’s Republic of China

^3^Department of Mechanics, Zhejiang University, Hangzhou 310027, People’s Republic of China

*Corresponding author

Email: seanwei@mail.xjtu.edu.cn

Contents

S1. Basic parameters of the resonator

S2. Implementing oscillation amplitude control through PLL

S3. Parameter identification

S4. Synchronization bandwidth of directly excited oscillator

**S1. Basic parameters of the resonator**

**Fig.S1 Schematic diagram of resonator structure**

**Table.S1 Dimensions and basic parameters of the resonators**

| Parameters | Value |
| --- | --- |
| Length of resonator (*l*) | 416 μm |
| Width of resonator (*w*) | 3.4 μm |
| Length of electrode (*l_d_*) | 215 μm |
| Width of resonator (*w_d_*) | 8 μm |
| Length of connection beam (*l_s_*) | 5 μm |
| Width of connection beam (*w_s_*) | 50 μm |
| Thickness of resonator (*h*) | 25 μm |
| Air gap (*g*) | 3 μm |
| Initial resistance (*R*) | 398.3 Ω |
| Density (*ρ*) | 2320 kg/m^3^ |
| Young's modulus (*E*) | 169 GPa |

**S2. Implementing oscillation amplitude control through PLL**

Phase lock loop was used to accurately control the amplitude of the oscillator and also obtained the quintic branch in Fig.1. The unstable solution of the resonator can be calibrated through such closed-loop approach, and it is more precisely compared to open-loop frequency sweeping^1^. The close loop test was performed as the following steps:1) After setting the experimental parameters and preheating the device for over 10 minutes, the forward frequency sweep was used to excite the resonator near the selected point; 2) Phase locked loop (Built in HF2LI -Zurich Instruments) was utilized to lock the phase;3) Manually changing the set point phase in PLL and recording the real time phase, amplitude and frequency, the desired amplitude point under a fixed excitation can be realized.

**Fig.S2 Schematic diagram of amplitude control in PLL.** The open loop test results of parametric excitation with *V_ac_*=550 mV and *V_dc_*=35 V.

**S3.** **Parameter** **identification**

**Fig.S3** **Parameter identification experiment setup and directly excited dynamic responses. a** The schematic diagram of capacitance detection method, the ends of the resonator were applied with ±10V, the left electrode was subjected to a DC voltage *V_dc_*=35V combined with AC voltage *V_ac_* from Lock-in amplifier HF2LI. The bias voltage *V_bais_* was set as 20V to detect the vibration amplitude. **b** The amplitude frequency curves with driving voltage *V_ac_* from 10 mV to 320 mV.

As Fig.S3 shows, the capacitance detection method is used to obtain the actual amplitude of the resonator. The adjustable capacitor *C_p_* was employed to eliminate the parasitic capacitance. The driving voltage *V_ac_* is changed from 10 mV to 320 mV to obtain the backbone characteristic. The cubic and quintic nonlinear stiffness of the resonator can be determined by analyzing the relationship between its frequency and peak amplitude. Similarly, the nonlinear damping of the resonator can be determined by analyzing the relationship between the driving force and peak amplitude^2^. The conversion between the true amplitude of the resonator and the readout voltage can be written as^3^:

$$Amp=\frac{\sqrt{2}g^{2}}{\omega_{0}\varepsilon_{0}AV_{bias}}\frac{V_{out}}{R_{f}}$$

Where *g* is the gap between fixed electrode and the resonator, *A* is the area between the plates facing each other. *ε*_0_ is the dielectric constant and *V_out_* is the measured amplitude from HF2LI. A resistor *R_f_* with a resistance value of 470 kΩ is used. The nonlinear vibration system can be described as

where *m_eff_* is the effective mode mass of the vibration mode which can be obtained in finite element analysis, *c* and *c_n_* is the linear and nonlinear damping in the system, respectively. *k*_1_, *k*_3_ ,*k*_5_ is the linear stiffness ,cubic nonlinear stiffness and quintic nonlinear stiffness respectively. The driving force *F*_0_ can be calculated by

$$F_{0}=\frac{2\varepsilon_{0}AV_{dc}V_{ac}}{g^{2}}$$

For simplification, the Eq. can be simplified as:

where the parameters in Eq. are

$$\mu=\frac{1}{Q},\eta=\frac{c_{n}}{m_{eff}\omega_{0}},\omega_{0}=\sqrt{\frac{k_{1}}{m_{eff}}},\beta=\frac{k_{3}}{m_{eff}\omega_{0}^{2}},\kappa=\frac{k_{5}}{m_{eff}\omega_{0}^{2}},f_{0}=\frac{F_{0}}{m_{eff}\omega_{0}^{2}},\Omega=\frac{\Omega_{d}}{\omega_{0}}$$

The approximate analytical solution of Eq. can be obtained by the method of multiple scales.

Considering steady-state vibration$A'(t)=\theta'\left(t\right)=0$ and the peak occurs for we obtain the backbone curve and the relationship between driving force and amplitude peak.

$$\Omega=\omega_{0}+\frac{3A^{2}\beta}{8\omega_{0}}+\frac{5A^{4}\kappa}{16\omega_{0}}$$

$$f_{0}=\mu A\omega_{0}+\frac{1}{4}\eta A^{3}\omega_{0}$$

Changing *V_ac_* from 10 mV to 320 mV and upward sweep the frequency, the relationship of peak amplitude and driving force can be obtained and shown in Fig.S2. Then, fitting Eq. and Eq., the nonlinear coefficient in Eq. can be determined.

**Fig.S4** Nonlinear coefficient of the vibration system. **a** The frequency of the resonator increases with amplitude under cubic nonlinear stiffness *k*_3_ and quintic nonlinear stiffness *k*_5_. The fitted *k*_3_ is 1.71×10^17^ N·m^-3^ and *k*_5_ is -7.83×10^28^ N·m^-5^. **b** The amplitude of the resonator increases nonlinearly with the increase of driving force. The fitted nonlinear damping *C_n_* is 4.8×10^5^ kg·m^-2^.

**S4. Synchronization bandwidth of directly excited oscillator**

With a perturbation signal, the close loop system can be modified as

Substituting the Van der Pol transformation *x*(*t*)=*A*(*t*)cos(*ϕ*(*t*)) and d*x*/d*t*=-*A*(*t*)Ωcos(*ϕ*(*t*)) into Eq., the following relationships are obtained.

Integrating the Eq. and averaging over a period, we obtain

where the $\Delta\omega=\frac{\omega_{0}^{2}-\Omega^{2}}{2\Omega}\approx\omega_{0}-\Omega$ and *ϑ*=*φ*-*θ*. The steady-state amplitude can be obtained by considering $A'(t)=0$ with the perturbation force *f_s_*=0.

With a weak perturbation force added, the amplitude can be assumed as *A*(*t*)=*R*+*εr*(*t*)+O(*ε*^2^), where *ε*=*f_s_*/*f*_0_≪1. Substituting the amplitude *A*(*t*) into Eq. and considering only the uncertainties caused by the disturbance, the expression for the perturbated amplitude *r*(t) of the disturbance can be obtained as:

Considering the steady motion and neglecting the high order terms, we obtain

Substituting *A*(*t*) into Eq., we obtain the expression for the phase difference after synchronization.

where the ΔΩ=Ω-Ω*_s_* and the synchronize interval is [Ω-Ω*_c_*,Ω+Ω*_c_*].

$$\Omega_{c}=-\frac{\cos\left(\vartheta\right)f_{s}}{2R\Omega}+\frac{3\beta Rf_{s}\sin(\vartheta)+5\kappa R^{3}f_{s}\sin(\vartheta)}{\Omega^{2}\left(4\mu+3\eta R^{2}\right)}$$

Therefore, the synchronization bandwidth under directly excitation is

$$B_{d}=2\sqrt{\left(\frac{f_{s}}{2R\Omega}\right)^{2}+\left(\frac{3\beta Rf_{s}+5\kappa R^{3}f_{s}}{4\mu\Omega^{2}+3\eta R^{2}\Omega^{2}}\right)^{2}}$$

**References**

1 Cuairan, M. T., Gieseler, J., Meyer, N. & Quidant, R. Precision Calibration of the Duffing Oscillator with Phase Control. *Phys Rev Lett* **128**, 213601, doi:10.1103/PhysRevLett.128.213601 (2022).

2 Kaisar, T., Lee, J., Li, D., Shaw, S. W. & Feng, P. X. Nonlinear Stiffness and Nonlinear Damping in Atomically Thin MoS(2) Nanomechanical Resonators. *Nano Lett*, doi:10.1021/acs.nanolett.2c02629 (2022).

3 Agarwal, M. *et al.* Scaling of amplitude-frequency-dependence nonlinearities in electrostatically transduced microresonators. *Journal of Applied Physics* **102**, doi:10.1063/1.2785018 (2007).
